# Fine-Tuning of Pten Localization and Phosphatase Activity Is Essential for Zebrafish Angiogenesis

**DOI:** 10.1371/journal.pone.0154771

**Published:** 2016-05-03

**Authors:** Miriam Stumpf, Sasja Blokzijl-Franke, Jeroen den Hertog

**Affiliations:** 1 Hubrecht Institute–Koninklijke Nederlandse Akademie van Wetenschappen (KNAW) and University Medical Center Utrecht, Utrecht, The Netherlands; 2 Institute of Biology Leiden, Leiden University, Leiden, The Netherlands; Institute of Cellular and Organismic Biology, TAIWAN

## Abstract

The lipid- and protein phosphatase PTEN is an essential tumor suppressor that is highly conserved among all higher eukaryotes. As an antagonist of the PI3K/Akt cell survival and proliferation pathway, it exerts its most prominent function at the cell membrane, but (PIP_3_-independent) functions of nuclear PTEN have been discovered as well. PTEN subcellular localization is tightly controlled by its protein conformation. In the closed conformation, PTEN localizes predominantly to the cytoplasm. Opening up of the conformation of PTEN exposes N-terminal and C-terminal regions of the protein that are required for both interaction with the cell membrane and translocation to the nucleus. Lack of Pten leads to hyperbranching of the intersegmental vessels during zebrafish embryogenesis, which is rescued by expression of exogenous Pten. Here, we observed that expression of mutant PTEN with an open conformation rescued the hyperbranching phenotype in *pten* double homozygous embryos and suppressed the increased p-Akt levels that are characteristic for embryos lacking Pten. In addition, in *pten* mutant and wild type embryos alike, open conformation PTEN induced stalled intersegmental vessels, which fail to connect with the dorsal longitudinal anastomotic vessel. Functional hyperactivity of open conformation PTEN in comparison to wild type PTEN seems to result predominantly from its enhanced recruitment to the cell membrane. Enhanced recruitment of phosphatase inactive mutants to the membrane did not induce the stalled vessel phenotype nor did it rescue the hyperbranching phenotype in *pten* double homozygous embryos, indicating that PTEN phosphatase activity is indispensable for its regulatory function during angiogenesis. Taken together, our data suggest that PTEN phosphatase activity needs to be carefully fine-tuned for normal embryogenesis and that the control of its subcellular localization is a key mechanism in this process.

## Introduction

PTEN is a major tumor suppressor that antagonizes the PI3K/Akt (also known as protein kinase B, PKB) pro-survival and -proliferation signaling pathway by dephosphorylating the lipid second messenger phosphatidylinositol (3, 4, 5)-trisphosphate (PIP_3_) to phosphatidylinositol (4,5)-bisphosphate (PIP_2_) [1]. In many cancer types, one or multiple components of this pathway can be found mutated. Upon loss of PTEN function, enhanced PIP_3_ signaling potentially drives cells into excessive proliferation and tumorigenesis by enhanced activation of the downstream effector, Akt [2,3]. Dephosphorylation of PIP_3_ is an event that occurs upon binding of PTEN to phospholipid-rich areas at the inner cell membrane [1,4]. There are other functions of PTEN, such as the regulation of DNA damage repair, genome stability, apoptosis, cell cycle progression or autophagy, that have been attributed to its nuclear localization [5–11].

During the past decade, it has become evident that controlling PTEN protein conformation and thereby its subcellular localization is a key regulatory mechanism to manage the variety of PTEN cellular functions. In its closed conformation, PTEN is predominantly cytoplasmic, while opening up its conformation results in re-location of PTEN to the cell membrane and the nucleus [8,12–15]. The conformational status of PTEN is predominantly regulated by post-translational modifications, especially by (de)-phosphorylation of the C-terminal tail, which alters the intramolecular interactions within PTEN [13,14,16,17]. PTEN is phosphorylated by multiple kinases, including GSK3, CK2 and ATM [11,18–20]. In the context of conformational changes, especially the phosphorylation by CK2 is an important event [19]. Phosphorylation on S370, S380, T382, T383 and S385 renders PTEN in its closed conformation, having the C-terminus folded back onto the N-terminus. Upon dephosphorylation of PTEN at these positions, the molecule adopts the open conformation, in which newly exposed side chains and amino acid motifs relocalize PTEN to either the cell membrane or the nucleus, depending on the cellular context [12,15,21]. Mutation of these five phosphorylation sites to alanines (S370A, S380A, T382A, T383A and S385A) mimics constitutive dephosphorylation at these sites and renders a PTEN mutant termed PTEN QMA or ‘open conformation PTEN’ [19,22,23]. One of the motifs that is potentially exposed upon opening of the PTEN protein conformation is the positively charged lysine-arginine-arginine (KRR) cluster within the N-terminus, which is involved in regulation of PTEN nuclear localization [24,25]. It has been shown that mono-ubiquitination of lysines within the N- and C- terminal tail, especially lysine 13, K13, regulates nuclear translocation of PTEN [26]. However, the regulation and mechanism of PTEN nuclear translocation as well as the identity of nuclear targets of PTEN remains to be determined definitively.

In this study, we investigated the functional *in vivo* consequences of expressing open conformation PTEN. We chose zebrafish as a model organism because of its high fecundity and its fantastic suitability for live cell imaging. Further, we have generated *pten* knockout zebrafish lines that facilitate functional characterization of Pten mutants. Like many other genes, *pten* has been duplicated during teleost evolution, resulting in two *pten* genes with redundant functions in zebrafish, *ptena* and *ptenb* [27,28]. By target selected gene inactivation (TSGI), we identified nonsense mutations in both *pten* genes, each resulting in a premature stop codon. We generated *ptena-/-* and *ptenb-/-* fish lines [28] that we incrossed to obtain *ptena+/-ptenb-/- and ptena-/-ptenb+/-* fish lines for our functional rescue assay. While their heterozygous and single homozygous siblings are viable and fertile, double homozygous embryos that lack all Pten activity develop a pleiotropic phenotype, characterized by massive edema formation, craniofacial defects, aberrant pigmentation and shorter body axis. Double homozygous *pten* mutant embryos are embryonic lethal around 5 days post fertilization (dpf) [28]. We previously studied the role of Pten in zebrafish angiogenesis and found that Pten is required for cell proliferation and migration during the formation of new blood vessels from the existing vasculature [29]. Zebrafish embryos that lack functional Pten are characterized by constitutively elevated pAkt and vegfaa levels, accompanied by enhanced angiogenesis that can be visualized from 3dpf onwards using confocal microscopy in *ptena-/-ptenb-/-* zebrafish embryos in the transgenic Tg(*kdrl*:*eGFP*) background[29,30]. Treatment with the PI3K-specific inhibitor LY294002, as well as microinjection of synthetic *pten* mRNA rescued the vasculature hyperbranching phenotype [29]. Recently, we reported that Pten lipid phosphatase activity is required for normal angiogenesis, whereas the complex process of embryonic development requires both lipid and protein phosphatase activity of Pten [31]. Here, we decided to screen functional mutants of Pten for their ability to rescue angiogenesis defects in Tg(kdrl:eGFP) zebrafish embryos that lack functional Pten.

PTEN is an important antagonist of pro-angiogenic signaling and likely exerts its role both in a cell autonomous way, activated by upstream Dll4/Notch signaling in stalk cells at the sprouting front to control cell proliferation [32], and in a non-autonomous way by suppressing vegfaa levels [29] in the surrounding tissue. Angiogenesis during embryonic development underlies the same, evolutionary conserved signaling pathways, as angiogenesis that is induced in adult ischemic tissues or by emerging tumors [33–35]. This is why PTEN is essentially important to suppress the angiogenic switch, a requisite for solid tumor growth and why lack of PTEN is associated with increased microvascular density in glioblastoma models [36] or gastric cancer biopsies [37]. Thus, a detailed understanding of how and when PTEN activity is fine-tuned during embryonic angiogenesis might potentially give new insights into its deregulation and therapeutic potential during tumor angiogenesis.

In this study, we investigated the rescue capacity of open conformation PTEN and found that its expression in *pten* double homozygous embryos rescued the characteristic hyperbranching vasculature phenotype and the increased levels of pAkt. Surprisingly, we also observed a significantly increased number of embryos exhibiting stalled intersegmental vessels that failed to connect with the dorsal longitudinal anastomotic vessel (DLAV). This phenotype was not observed upon microinjection of an equal dose of wild type PTEN mRNA. Further experiments suggest that specifically the enhanced membrane localization of PTEN induced the stalled vessels defect. We demonstrate that enhanced membrane localization of phosphatase active PTEN dramatically increased its biological function in suppression of angiogenic sprouting. Tight temporal and spatial regulation of PTEN activity during angiogenesis may therefore play an essential role.

## Materials and Methods

### Ethics statement

All procedures involving experimental animals described here were approved by the local animal experiments committee (Koninklijke Nederlandse Akademie van Wetenschappen-Dierexperimenten commissie KNAW-DEC protocol HI12.0701) and performed according to local guidelines and policies in compliance with national and European law.

### Fish line

Zebrafish were maintained and the embryos were staged according to standard protocols [38]. The lines tg(*H2A-eGFP*) and tg(*kdrl*:*eGFP*) have been previously described [39,40]. The *ptena-/-* and *ptenb* -/- zebrafish lines were created by target-selected gene inactivation (TSGI). Both the *ptena*^*hu1864*^ and the *ptenb*^*hu1435*^ allele result from non-sense mutations that lead to a premature stop codon upstream of the catalytic site [28].

### Constructs, mRNA synthesis and Micro-injections

The PTEN-mCherry and Ptenb-mCherry fusion constructs were obtained by PCR amplification of *ptenb* or *PTEN*, respectively, from the vectors described in [28]. The PTEN-mCherry QMA construct was obtained by amplification of PTEN QMA [22]. All PCR products were subsequently ligated into the pCS2+mCherry vector. The point mutations in the PTP domain and the N-terminus were introduced by site-directed mutagenesis. The constructs were linearized with NotI and to synthesize 5’ capped sense mRNA, the mMessage mMachine SP6 kit (Ambion) was used. mRNA injections were performed at the one-cell stage as described using a total of 300 pg of mRNA.

### Genotyping

Zebrafish embryos (4 dpf) were anesthesized with 16mg/ml 3-amino benzoic acid ethylesther (MS-222) and cut in half (just below the yolk sack extension). The tail was lysed for genotyping and the head/trunk region was subjected to a protein lysis protocol for subsequent immunoblotting (see below). For genotyping, the genomic target sequence was amplified by two subsequent tilling PCRs, using the following primer pairs: Tilling 1: Ptena and Ptenb #1 and #4. Tilling 2: Ptena and Ptenb #2 and #3. For sequencing, diluted Tilling2 product and primer Ptena #2 or Ptenb #3 was submitted to Macrogen. The sequencing data was analyzed with Lasergene software.

Primer sequences:

Ptena #1 GCGCTAGTTTCTTGTTTAGATTG

Ptenb #1 AAAGAACAGAAATCaCAGTTCCA

Ptena #2 TGTTAACCTGGTGTACAGTGC

Ptenb #2 TGTTGAGCTTTTGTTGGATGA

Ptena #3 TGGGCAAAATTAAAGAGACC

Ptenb #3 TGCCAAAACCAACAGAACAA

Ptena #4 CAGACTATTATTTCCCCCAAAC

Ptenb #4 TGCTTAGAACTTTGCACCAA

### Immunoblotting

The trunks of 4 dpf zebrafish embryos were isolated (see above) and lysed in 25mM HEPES (pH7,4), 125mM NaCl, 0,25% Deoxycholate, 10mM MgCl_2_, 1mM EDTA, 1% Triton X-100, 10% Glycerol buffer containing proteinase and phosphatase inhibitors. Samples were lysed for 30min and subsequently subjected to sonication with the Bioruptor (settings: high intensity, 15min, 1min on/off cycles). Samples were run on a 10% SDS-PAGE gel, transferred to a PVDF membrane and stained with Coomassie Blue to verify equal loading. The blots were probed with antibodies specific to pAkt (1:2000), Akt (1:1000) (all Cell Signaling) and Tubulin (1:3000); (Calbiochem). For signal detection, enhanced chemiluminescence (Thermo Scientific kit) was diluted 1:2 in home- made ECL.

### Confocal microscopy and Analysis

Zebrafish embryos were anesthetized at 3dpf and laterally mounted on glass bottom dishes (Greiner bio one) in 0.5% agarose (type V, Sigma Aldrich) in E3 embryo medium containing 16mg/ml 3-amino benzoic acid ethylesther (MS-222) to block contractile movements. Confocal microscopy was performed using a Leica TCS SPE, 20x objective. Z-stacks (step size 2μm) of the vasculature in the tail region, right below the yolk sack extension, were acquired for every embryo at 28.5°C. Image J software (http://rsb.info.nih.gov./ij/) was used to generate z-projection images of the zebrafish embryonic vasculature.

### Statistics

Significance of the frequency of occurrence of the “hyperbranching” and “stalled vessel” phenotypes in the different experimental conditions compared to the non-injected control was assessed using two-tailed Fisher’s exact test. Results were considered significant when p<0.05 (p values * p<0.05, ** p<0.01, *** p<0.001.)

## Results

### Open conformation of PTEN enhances both its membrane and nuclear localization *in vivo*

**Fig 1:**
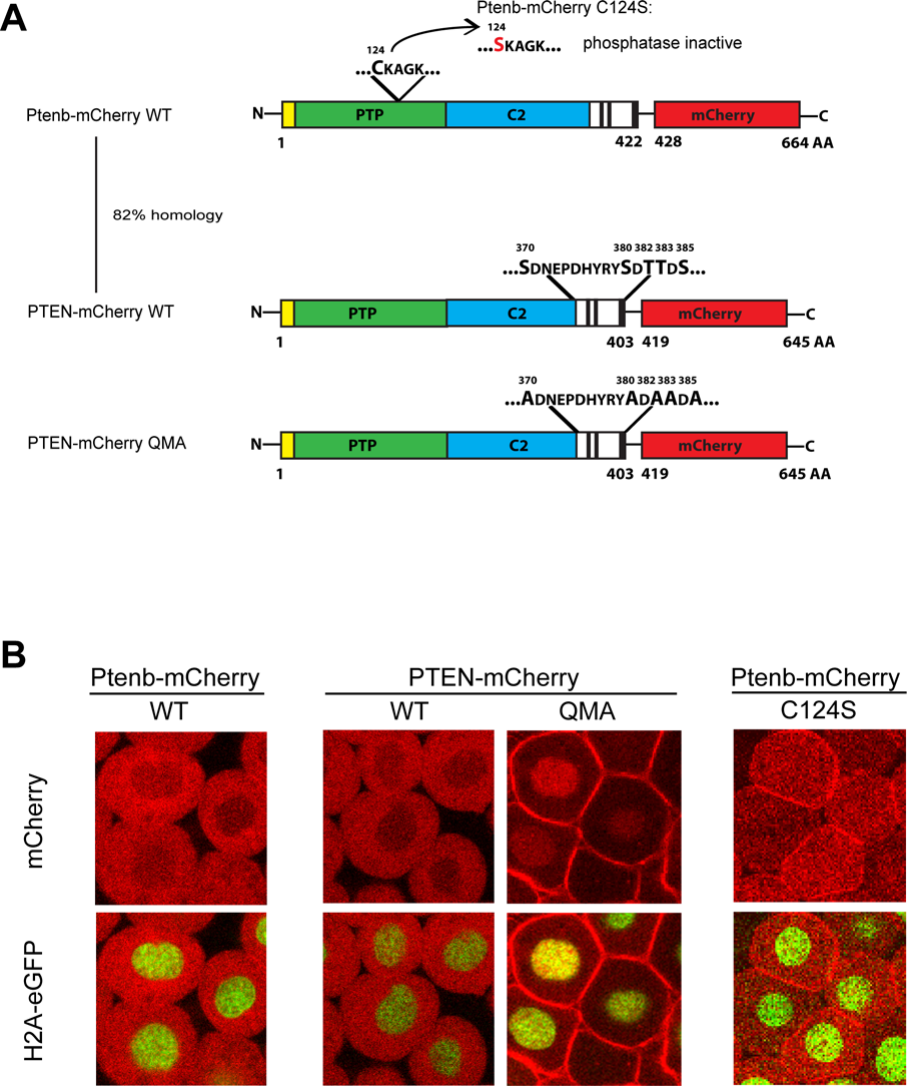
PTEN QMA localizes to the cell membrane and nucleus in zebrafish embryos. **(A)** Wild type Ptenb (long splicing variant, 422 amino acids) and human PTEN share a homology of 82%, both consisting of an N-Terminus (yellow), the PTP-domain (green), the C2 domain (blue) and the C-terminus (black and white). Mutation of the catalytic cysteine 124 to serine, C124S, results in catalytically inactive Ptenb. PTEN QMA contains five point mutations in its C-terminal phosphorylation sites, resulting in an open conformation of PTEN [19]. The red fluorescent protein mCherry (red) is C-terminally tagged in frame to all constructs used in these experiments. **(B)** Subcellular localization of Ptenb-mCherry, PTEN-mCherry, PTEN-mCherry QMA and Ptenb-mCherry C124S was assessed in tg*(H2A-eGFP)* zebrafish embryos, microinjected with 300pg synthetic mRNA at the one-cell stage and submitted to confocal live imaging at 6hpf. Top panels: mCherry expression; bottom panels: overlay of mCherry expression with H2A-GFP, which localizes to the nucleus.

Previously, we have used the ability of Pten mutants to rescue the hyperbranching phenotype in zebrafish embryos lacking enodgenous Pten as a read-out for Pten function [31]. Modulation of the conformation of PTEN is an established regulatory mechanism. Here, we investigated the consequences of expressing mutant PTEN with constitutively open conformation in live zebrafish embryos. Wild type zebrafish Ptenb-mCherry and phosphatase-inactive mutant Ptenb-mCherry C124S (Fig 1A) were used as positive and negative controls, respectively. Constructs encoding human PTEN-mCherry, which is highly homologous (81%) to zebrafish Ptenb and open conformation PTEN QMA-mCherry were derived (Fig 1A). PTEN QMA contains five mutations of phosphorylation sites (S370A, S380A, T382A, T383A, S385A). All five phosphorylation sites and surrounding sequences are absolutely conserved in zebrafish Ptena as well as Ptenb. Nevertheless, we chose to use human PTEN and PTEN QMA for these experiments, to exclude the possibility that the conformational change in PTEN QMA was not reproduced in mutant zebrafish Ptenb with the homologous five substitutions, due to small differences in the sequence of Ptenb away from the five phosphorylation sites.

To characterize and compare subcellular localizations of the zebrafish and human PTEN-mCherry fusions, we microinjected Tg(*H2A-eGFP*) zebrafish embryos at the one-cell stage with synthetic mRNA encoding the above mentioned constructs and performed confocal live imaging on the zebrafish embryos at 6hpf (Fig 1B). Ptenb-mCherry and PTEN-mCherry both localized predominantly to the cytoplasm with only weak fluorescence in the nucleus. Ptenb-mCherry C124S was evenly distributed over cytoplasm and nucleus and showed clearly enhanced membrane localization. PTEN QMA-mCherry localized away from the cytoplasm, towards the cell membrane and to the nucleus, consistent with the open conformation of PTEN QMA.

### PTEN QMA rescues hyperbranching in *pten* mutants and induces stalled intersegmental vessels

**Fig 2:**
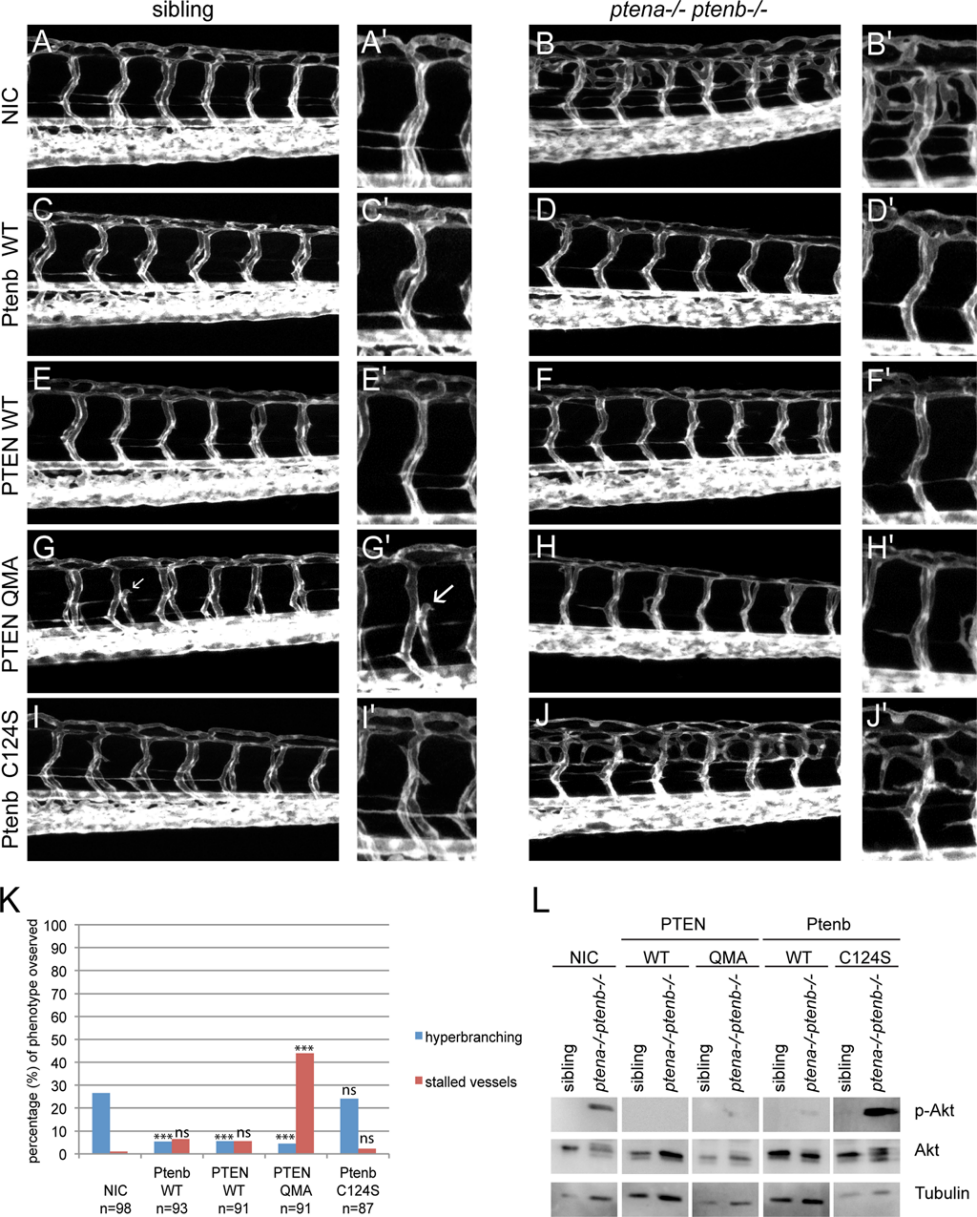
Ptenb, PTEN and PTEN QMA, but not Ptenb C124S rescued the hyperbranching vasculature phenotype. **(A-J)** Zebrafish embryos from a tg(*kdrl*:*eGFP*) *ptena+/- ptenb-/-* incross were microinjected at the one-cell stage with 300 pg synthetic mRNA encoding wild type PTEN-mCherry, PTEN-mCherry QMA, Ptenb-mCherry WT or Ptenb-mCherry C124S. At 3dpf the embryos were analyzed for the hyperbranching vessel phenotype by confocal live- imaging on a Leica TCS-SPE microscope (anterior to the left, 20x objective, 2μm z-stacks). Pictures show the trunk region distal from the urogenital opening of representative, genotyped embryos. Non-injected control embryos (control) were included for reference. **(A’-J’)** A close-up is added on the right side of each image. **(G)** Remarkably, we found a second phenotype in the PTEN-mCherry QMA injected embryos, consisting of a significantly increased number of lacking or stalled intersegmental vessels (indicated with white arrows). **(K)** Quantification of the embryos showing the typical *ptena*-/-*ptenb*-/- hyperbranching vessel phenotype at 3 dpf (blue bars). In the non-injected control (NIC), approximately 25% of the embryos showed the characteristic phenotype (Mendelian segregation). The percentage of embryos showing the stalled vessel phenotype at 3dpf is also indicated (red bars). The statistical significance of each of the conditions compared to the non-injected control was determined using two-tailed Fisher’s exact test and is indicated in the bar graph (ns = not significant, * = p-value < 0.05, ** = p-value < 0.01, *** = p-value < 0.001). **(L)** Embryos from a *ptena+/-ptenb-/-* incross were microinjected at the one-cell stage with wild type PTEN, PTEN QMA, wild type Ptenb or Ptenb C124S. At 4dpf, single embryos were cut in half. The trunk region was used for genotyping and the anterior half was lysed and processed for immunoblotting. Lysates from siblings and *ptena-/-ptenb-/-* embryos were run side by side on gels and blotted. The membranes were probed with phosphospecific anti-pAkt antibody (directed against pSer473), stripped and probed with Akt-specific antibody, stripped and probed with a Tubulin-specific antibody as a loading control. Representative blots are shown.

Angiogenesis, the formation of new blood vessels from the existing vasculature, is a process tightly controlled by PI3K signaling and therefore a good read-out to evaluate the rescue capacity of PTEN mutants in *pten* double homozygous zebrafish embryos. In order to functionally characterize PTEN and PTEN QMA in angiogenesis, we microinjected embryos from a Tg(*kdrl*:*eGFP*) *ptena+/-ptenb-/-* incross at the one-cell stage with synthetic mRNA, encoding Ptenb-mCherry, PTEN-mCherry or PTEN-mCherry QMA. At 3dpf, we monitored the vasculature by confocal live imaging (Fig 2A–2J). According to Mendelian law, 25% of the offspring of a *ptena+/-ptenb-/-* incross was expected to be double homozygous and develop the hyperbranching phenotype (Fig 2K). This percentage was reflected in the non-injected control (NIC) embryos. Expression of Ptenb, PTEN or PTEN QMA significantly decreased the percentage of embryos with the characteristic hyperbranching phenotype (Fig 2D, 2F, 2H and 2K), whereas the phosphatase inactive Ptenb C124S did not have this effect (Fig 2J and 2K). Surprisingly, we discovered a second phenotype in the *ptena-/-ptenb-/-* embryos and siblings expressing PTEN QMA (Fig 2G’). This phenotype consisted of the occurrence of intersegmental vessels (ISV) that did not connect to the dorsal longitudinal anastomotic vessel (DLAV) but instead stalled somewhere along the way. This stalled vessel phenotype might be caused by excessive activity of PTEN towards PIP_3_, leading to diminished VEGFR signaling. In order to test this hypothesis, we assessed the levels of phosphorylated Akt, p-Akt (Fig 2L). Our data confirmed that PTEN QMA, as well as PTEN WT, reduced the elevated p-Akt levels in 4dpf *ptena-/-ptenb-/-* embryos at least as effectively as Ptenb WT. Ptenb C124S lacks phosphatase activity and did not reduce p-Akt levels.

### Lysine 13 mutations modulate Ptenb subcellular localization

**Fig 3:**
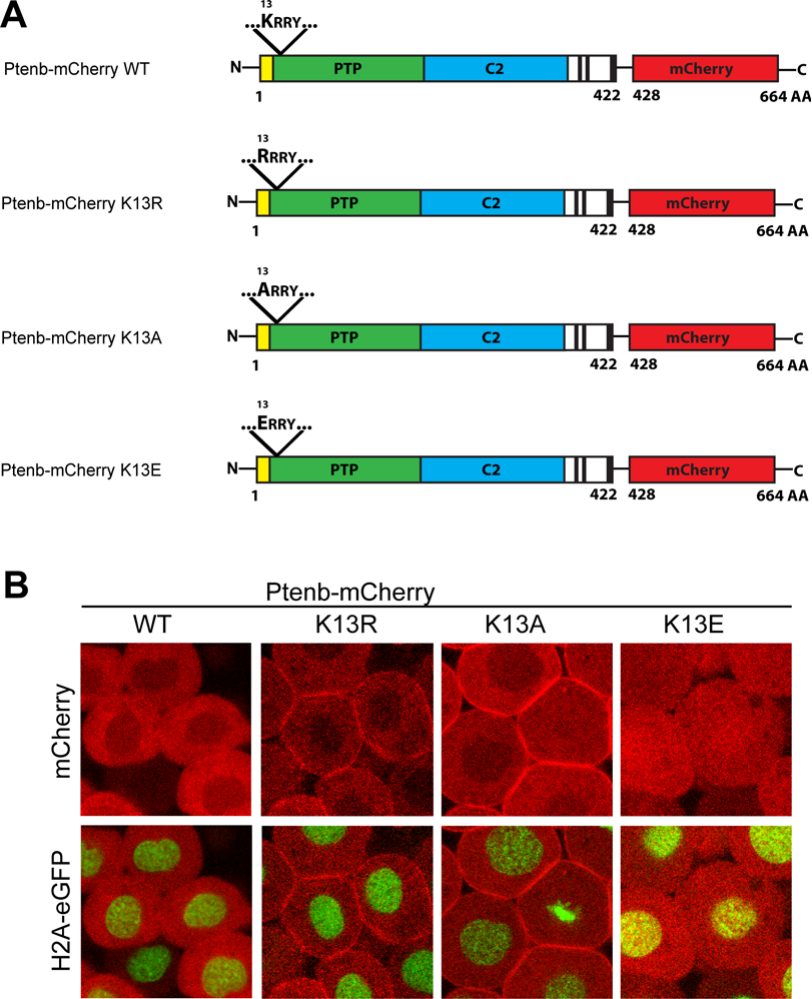
Mutation of Pten K13 alters Pten subcellular localization in 6hpf zebrafish embryos. **(A)** Point mutations were introduced into the N-terminus of Ptenb (long splicing variant, 422 amino acids) via site-directed mutagenesis. K13 was replaced by either arginine (K13R, charge conserving), alanine (K13A, change to neutral charge) or glutamate (K13E, charge inverting) to address the importance of lysine 13 for the subcellular localization of Pten. The red fluorescent protein mCherry (red) is C-terminally tagged in frame to all constructs used in these experiments. **(B)** Subcellular localization of Ptenb-mCherry K13R, K13A and K13E was assessed in tg*(H2A-eGFP)* zebrafish embryos, microinjected with 300pg synthetic mRNA and submitted to confocal live imaging at 6hpf. Top panels: mCherry expression; bottom panels: overlay of mCherry expression with H2A-GFP, which localizes to the nucleus.

In all other experimental conditions, the number of fish displaying the stalled vessel phenotype was remarkably low, indicating that it is specifically caused by microinjection of PTEN in its open conformation, PTEN QMA. Next, we investigated whether it was rather the nuclear localization or the membrane accumulation of open conformation PTEN that induced the stalled vessel phenotype and whether the phosphatase activity of PTEN played a crucial role in this process. Some mutations in the N-terminus of Pten, such as mutations of the putative mono-ubiquitination site lysine 13 (K13) (Fig 3A), alter its subcellular localization and shift it either towards the cell membrane (Ptenb-mCherry K13R, Ptenb-mCherry K13A) or to the nucleus (Ptenb-mCherry K13E) (Fig 3B). We assessed the altered subcellular localization of these constructs by microinjecting Tg(*H2A-eGFP*) zebrafish embryos with the synthetic mRNAs at the one-cell stage and at 6hpf, the embryos were screened for Pten subcellular localization by confocal live imaging. The charge conserving mutation of Ptenb-mCherry K13R enhanced the accumulation of Ptenb at the cell membrane. The same localization pattern was observed for the positive to neutral charge mutation of Ptenb-mCherry K13A. Mutation of positively charged lysine 13 to negatively charged glutamic acid, Ptenb-mCherry K13E, resulted in accumulation in the nucleus (Fig 3B). These Ptenb mutants allowed us to assess whether subcellular localization affected Pten function.

### Accumulation of phosphatase-active Pten at the membrane induces stalled intersegmental vessels

**Fig 4:**
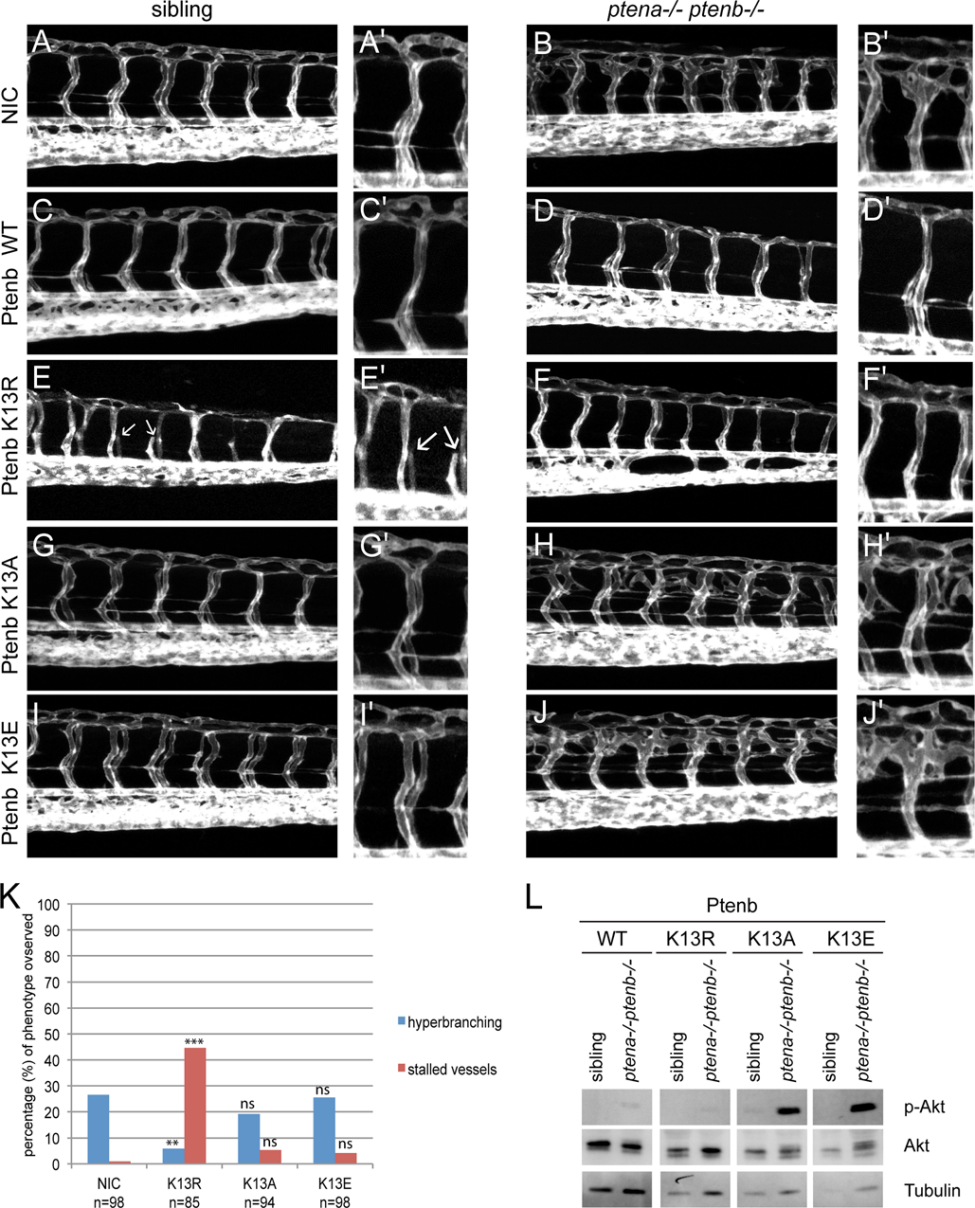
Enhanced membrane accumulation of phosphatase-active Pten induces stalled intersegmental vessels. **(A-J)** Zebrafish embryos from a tg(*kdrl*:*eGFP) ptena+/-ptenb-/-* incross were microinjected at the one-cell stage with 300 pg synthetic mRNA encoding either Ptenb-mCherry WT, K13R, K13A or K13E. At 3dpf the embryos were analyzed for the hyperbranching vessel phenotype by confocal live imaging on a Leica TCS-SPE microscope (anterior to the left, 20x objective, 2μm z-stacks). Pictures show the trunk region distal from the urogenital opening of representative, genotyped embryos. Non-injected control embryos (NIC) were included for reference. **(A’-J’)** A close-up is added on the right side of each image. Stalled vessels are indicated with white arrows. **(K)** Quantification of the embryos expressing the indicated Ptenb mutants, showing the typical *ptena*-/-*ptenb*-/- hyperbranching vessel phenotype (blue bars) or the stalled vessel phenotype (red bars) at 3dpf. In the non-injected control (NIC), approximately 25% of the embryos showed the characteristic hyperbranching phenotype (Mendelian segregation). The statistical significance of each of the conditions compared to the non-injected control was determined using two-tailed Fisher’s exact test and is indicated in the bar graph (ns = not significant, * = p-value < 0.05, ** = p-value < 0.01, *** = p-value < 0.001). **(L)** Embryos from a *ptena+/-ptenb-/-* incross were microinjected at the one-cell stage with wild type Ptenb or Ptenb K13 mutants as indicated. At 4dpf, single embryos were cut in half. The trunk region was used for genotyping and the anterior half was lysed and processed for immunoblotting. Lysates from siblings and *ptena-/-ptenb-/-* embryos were run side by side on gels and blotted. The membranes were probed with phosphospecific anti-pAkt antibody (directed against pSer473), stripped and probed with Akt-specific antibody, stripped and probed with Tubulin-specific antibody as a loading control. Representative blots are shown.

We investigated whether subcellular localization of Ptenb affected the rescue capacity of the hyperbranching defect on the one hand and induced stalled vessels on the other. To this end, we microinjected Tg(*kdrl*:*eGFP*) *ptena+/-ptenb-/-* incross embryos at the one-cell stage with synthetic mRNA, encoding Ptenb WT, Ptenb K13R, K13A or K13E. At 3dpf, we analyzed the embryonic vasculature by confocal live imaging (Fig 4A–4J). We detected the characteristic hyperbranching vessel phenotype in about 25% of the Ptenb-mCherry K13E and Ptenb-mCherry K13A injected embryos, like in the non-injected control (NIC) embryos (Fig 4K). This finding is consistent with published data that PTEN K13E and K13A mutants exhibit diminished or even abolished phosphatase activity [12]. Ptenb-mCherry K13R on the other hand significantly rescued the hyperbranching phenotype of *pten* double homozygous embryos (Fig 4F and 4K). Furthermore, similar to the PTEN QMA injected embryos (Fig 2G), we observed a dramatic increase of the amount of stalled vessel phenotypes in the Ptenb-mCherry K13R injected embryos. Besides Ptenb-mCherry K13R, none of the other lysine 13 mutants significantly induced the stalled vessel phenotype. At 4dpf, the embryos were genotyped, lysed for immunoblotting and probed for p-Akt-levels (Fig 4L). Ptenb-mCherry WT, as well as Ptenb-mCherry K13R drastically decreased p-Akt levels in *ptena-/-ptenb-/-* zebrafish embryos, whereas neither Ptenb-mCherry K13A or K13E had this effect. A trivial explanation for the lack of effects of Ptenb K13A and K13E might be that these proteins are not stable and are rapidly degraded. Unfortunately, due to low expression levels of all mutant Pten-mCherry proteins, we were not able to monitor protein expression in the zebrafish embryos. However, transfection of the constructs encoding the (mutant) Pten proteins in human embryonic kidney 293 cells revealed similar expression levels of all mutant Pten proteins, suggesting that there are no big differences in protein stability (S1 Fig). Therefore, we infer in concordance with data obtained for human PTEN [12], that zebrafish Ptenb K13E and K13A both significantly lack phosphatase activity towards phospholipids. The charge conserving K13R mutation, which caused enhanced accumulation of Ptenb at the cell membrane but not in the nucleus, induced stalled vessels with a similar incidence as PTEN QMA. Increased accumulation of phosphatase inactive Pten at the cell membrane, such as Ptenb-mCherry C124S or K13A, however, did not result in this phenotype. Altogether this led us to conclude that it is the excessive membrane accumulation of phosphatase active PTEN/Ptenb that caused the stalled intersegmental vessels during angiogenesis.

### Dominant effects of Ptenb K13R and PTEN QMA on angiogenesis in wild type fish

**Fig 5:**
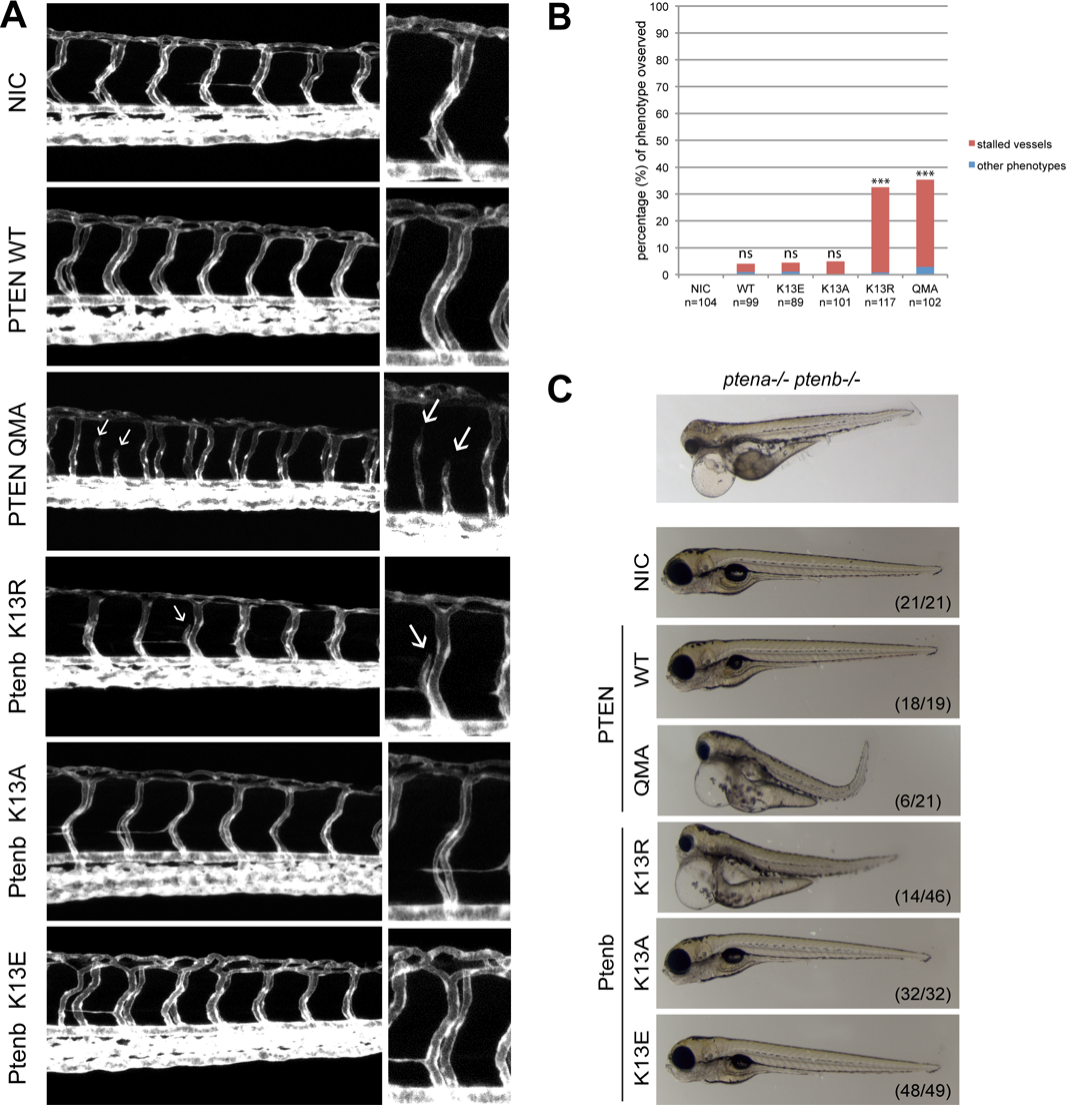
Ptenb K13R and PTEN QMA induce stalled vessels and developmental defects in wild type embryos. **(A)** Tg*(kdrl*:*eGFP)* zebrafish embryos were microinjected at the one-cell stage with 300 pg synthetic mRNA encoding either PTEN-mCherrry WT, PTEN-mCherry QMA, Ptenb-mcherry K13R, Ptenb-mCherry K13A or Ptenb-mCherry K13E. At 3dpf the embryos were analyzed for the hyperbranching or stalled vessel phenotype by confocal live- imaging using a Leica TCS-SPE microscope (anterior to the left, 20x objective, 2μm z-stacks). Pictures show the trunk region distal to the urogenital opening of representative embryos; non-injected control embryos (NIC) were included for reference. Stalled vessels are indicated with white arrows. **(B)** Quantification of the number of embryos showing either stalled intersegmental vessels (red bars) or other phenotypes, such as hyperbranching (blue bars) (percentage of total number of embryos). The statistical significance was determined using two-tailed Fisher’s Exact test. (ns = not significant, * = p-value < 0.05, ** = p-value < 0.01, *** = p-value < 0.001). **(C)** At 4dpf, brightfield images of single embryos were taken to assess gross morphological defects in response to expression of PTEN, Ptenb or mutants. A picture of a mutant *ptena-/-ptenb-/-* embryo (top panel) is provided as a reference. Note the massive edemas, short body axis and craniofacial abnormalities in the *ptena-/-ptenb-/-* embryo as well as the embryos expressing PTEN QMA and Ptenb K13R. The numbers in the bottom right corner represent the number of embryos showing the depicted phenotype/ total number of embryos.

To address the question whether excessive membrane accumulation of phosphatase active PTEN might induce angiogenesis defects in *pten* wild type fish, we microinjected Tg(*kdrl*:*eGFP*) embryos at the one-cell stage with synthetic RNA encoding PTEN-mCherry, PTEN-mCherry QMA or Ptenb-mCherry, K13R, K13A or K13E and subsequently performed confocal live imaging of their trunk vasculature (Fig 5A). As expected, the non-injected control (NIC) embryos did not display any of the two vasculature phenotypes, whereas microinjection of synthetic Pten mRNA occasionally induced stalled vessels in a small subset of embryos (around 3–5%). This background however is not statistically significant, thus neither Ptenb-mCherry, or Ptenb-mCherry K13E or K13A induced significant angiogenesis defects. PTEN QMA and Ptenb K13R, both with enhanced membrane localization, on the other hand induced stalled or even missing intersegmental vessels in 31–33% of the injected embryos (Fig 5A and 5B). We conclude that localization of phosphatase-active Pten to the cell membrane induced stalled vessel defects.

### Morphological defects upon expression of membrane-localized active Pten

The overall morphology of embryos expressing (mutant) Ptenb was assessed by brightfield microscopy at 4dpf. We chose this time point because it is the developmental stage at which the pleiotropic phenotype of *ptena-/-ptenb-/-* embryos becomes apparent. Surprisingly, Ptenb K13R and PTEN QMA induced phenotypes similar to the *pten* double homozygous phenotype (Fig 5C) [28,31]. Common features of the phenotypes include massive edemas, craniofacial defects, shorter body axis and aberrant pigmentation. Expression of wild type PTEN or mutant Ptenb K13A and Ptenb K13E did not induce morphological defects (Fig 5C). These data indicate that expression of mutant Pten with enhanced function phenocopied embryos that lack functional Pten.

## Discussion

PTEN subcellular localization is regulated by protein conformation, which in turn is largely dependent on the phosphorylation status of the PTEN C-terminal tail, thus regulating PTEN membrane and nuclear localization [23]. PTEN catalytic activity is also regulated by conformational changes within the protein’s tertiary structure [12,41]. We were interested in studying the physiological consequences of expressing open conformation PTEN, PTEN QMA, in a multicellular organism. Zebrafish is a good model for our purposes, due to the extra-uterine development and the transparency of the embryos [30]. The similar localization of both zebrafish and human PTEN in our model was indicative of the similarity of the two highly conserved homologues at the functional level. Therefore, we further characterized the human open conformation PTEN QMA in our functional rescue assay, taking along PTEN WT, Ptenb WT and Ptenb subcellular localization mutants. Our results confirmed that human PTEN compensates for lack of zebrafish Pten during angiogenesis in *pten* double homozygous embryos. Remarkably, microinjection of phosphatase active Pten with enhanced membrane recruitment, like PTEN QMA and Ptenb K13R, rescued the hyperbranching phenotype and in addition induced a stalled vessel phenotype. Since the hyperbranching phenotype emerges upon lack of Pten, we hypothesized that the stalled vessel phenotype might be provoked by enhanced Pten function. This enhanced Pten function might be a consequence of the increased recruitment to the cell membrane, a feature that both PTEN QMA and K13R share. It has been previously reported that PTEN K13R displayed an increased ability to counteract PI3K activity in yeast [24]. To date, only few studies have focused on the effect of overexpression of wild type PTEN. For instance, expression of wild type PTEN [42] or the splice variant, PTEN-L, *in vivo* have been reported, but no defects in angiogenesis or other serious adverse effects were reported in these models. Also in our study, overexpression of wild type PTEN or Ptenb did not increase the occurrence of stalled blood vessels at 3dpf. Lack of Pten and its consequences on angiogenesis, by contrast, have been more extensively studied *in vivo* and have been related to increased microvascular density and VEGF expression in gastric cancer biopsies [37]. The hyperbranching phenotype in developing zebrafish embryos is associated with increased PIP_3_ signaling, Akt signaling and *vegfaa* expression [29]. A recent study has further unraveled that Notch signaling induces enhanced transcription levels of Pten to suppress proliferation of the stalk cells within the sprouting front during angiogenesis in a cell-autonomous manner [32]. Moreover, that gain and loss of PTEN induces similar proliferation phenotypes in postnatal mouse retinas is consistent with our observations that zebrafish embryos that express mutant Pten or lack functional Pten display similar morphological defects. We believe that both the cell-autonomous and the paracrine functions of Pten are likely underlying the two opposing phenotypes, hyperbranching and stalled vessels. Membrane-associated, phosphatase active Pten may be hyperfunctional due to an increased PIP_3_ dephosphorylation rate, leading to decreased downstream Akt signaling and hence suppression of endothelial cell proliferation and migration, as well as reduced *vegfaa* transcription and secretion in the surrounding stroma cells. Serra et al. [32] further discovered that Pten exerts its anti-proliferative functions in angiogenesis by facilitating the APC/C-Fzr1/Cdh1 complex activity in the nucleus, independently of its phosphatase activity. Our results suggest that this function of Pten has a minor role during the suppression of pro-angiogenic signals, since Ptenb K13E, a phosphatase inactive mutant, shows a shift in subcellular localization pattern towards the nucleus, yet, did not rescue the hyperbranching phenotype in *pten* double homozygous embryos. However, we cannot completely rule out that enhanced nuclear localization of PTEN QMA contributes to the stalled vessel phenotype. Altogether, our results indicate that Pten K13R and PTEN QMA localize to the cell membrane and are potent and dominant inhibitors of Akt signaling. The enhanced membrane localization of Pten K13R and PTEN QMA could potentially be caused by an increased substrate binding affinity or an altered binding mechanism of both mutants in comparison to wild type PTEN or Ptenb. It has been recently discovered that the catalytic kinetics of PTEN-L and PTEN greatly differ from each other. Whereas PTEN dephosphorylates PIP3 in a “hopping” mode that allows for the diffusion between separate membranes, PTEN-L seems to dephosphorylate PIP3 in a “scooting” mode [43]. A similar change in membrane binding kinetics could be underlying the potential functional hyperactivity of PTEN QMA and Ptenb K13R. It is also likely that these two PTEN mutants deregulate other signaling pathways, for example signal transduction via focal adhesion kinase (FAK) [44–46]. Increased PTEN functionality may have direct or indirect effects on FAK, either by dephosphorylation of FAK by PTEN, or by transcriptional downregulation of FAK via the PI3K/Akt/NFĸB-axis [47], respectively. In either case, suppression of FAK activity would inhibit proliferation, migration, invasion and tube formation of vascular endothelial cells into the surrounding tissue during angiogenesis [48–50]. From our experiments, we conclude that PTEN function and the level of PIP3 signaling need to be tightly controlled for normal embryonic angiogenesis.

## Supporting Information

S1 Fig Similar expression levels of PTEN mutants. Human embryonic kidney 293 cells were transfected with empty vector, CMV-promoter driven expression vectors for human PTEN, PTEN QMA, a deletion mutant of PTEN lacking the N-terminal 16 residues (not relevant here), PTEN K13E, PTEN K13R and PTEN K13A. The cells were lysed and the lysates were run on an SDS-PAGE gel. The gels were blotted and the blots were probed with PTEN-specific antibodies and developed using enhanced chemiluminescence. Coomassie staining of the blot is provided as a loading control. Similar levels of (mutant) PTEN protein were detected, suggesting there are no major differences in stability between the mutant PTEN proteins.
(TIF)
