# Supplementary figures and images for: Fine-Tuning of Pten Localization and Phosphatase Activity Is Essential for Zebrafish Angiogenesis
Source: PLoS One. 2016 May 3;11(5):e0154771. doi: 10.1371/journal.pone.0154771 (PMC4854392; doi:10.1371/journal.pone.0154771)

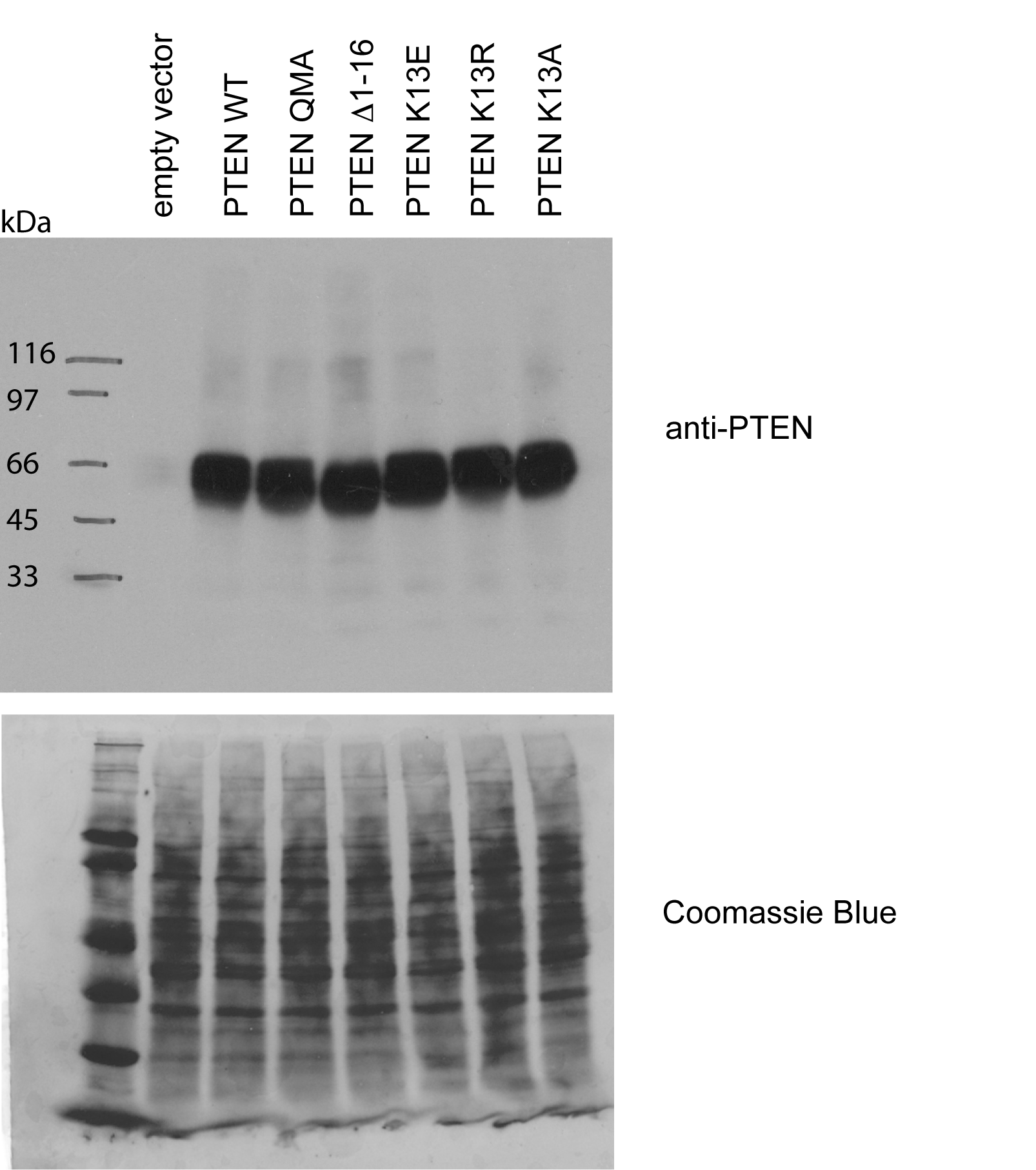

Supplement: S1 Fig — Human embryonic kidney 293 cells were transfected with empty vector, CMV-promoter driven expression vectors for human PTEN, PTEN QMA, a deletion mutant of PTEN lacking the N-terminal 16 residues (not relevant here), PTEN K13E, PTEN K13R and PTEN K13A. The cells were lysed and the lysates were run on an SDS-PAGE gel. The gels were blotted and the blots were probed with PTEN-specific antibodies and developed using enhanced chemiluminescence. Coomassie staining of the blot is provided as a loading control. Similar levels of (mutant) PTEN protein were detected, suggesting there are no major differences in stability between the mutant PTEN proteins. (TIF) [file pone.0154771.s001.tif]
